# Drought-tolerant rhizobacterial consortia enhance grapevine growth and tolerance to water deficit

**DOI:** 10.3389/fpls.2026.1735733

**Published:** 2026-02-06

**Authors:** Ginevra Canavera, Gabriele Bellotti, Harsh Tiwari, Tommaso Frioni, Edoardo Puglisi

**Affiliations:** 1 Department of Sustainable Food Process, Università Cattolica del Sacro Cuore, Piacenza, Italy; 2 Department of Sustainable Crop Production, Università Cattolica del Sacro Cuore, Piacenza, Italy; 3 National Research Centre for Agricultural Technologies (Agritech Foundation, PNRR M4C2), Naples, Italy

**Keywords:** grapevine, microbial biostimulants, plant adaptation and drought stress tolerance, plant growth-promoting rhizobacteria, rhizosphere microbiome, whole-genome sequencing

## Abstract

In both natural ecosystems and in agroecosystems, Plant Growth-Promoting Rhizobacteria (PGPR) significantly contribute to plant development and stress mitigation through diverse mechanisms. In recent times, their application as microbial biostimulants (MBs) has gained attention, particularly for alleviating drought stress, which increasingly threatens grapevine productivity in both modern and traditional wine-growing regions. Despite this interest, functionally validated and biosafe PGPR consortia specifically tailored for grapevine remain limited. This study isolated drought-tolerant PGPRs from grapevine rhizosphere cultivated under contrasting environmental conditions and experiencing midday leaf water potentials between −1.3 and −1.4 MPa. The isolates were ranked according to their Plant Growth-Promoting Traits (PGPTs), and whole-genome sequencing was performed on the nine most promising strains to evaluate their functional potential and biosafety. Such functional traits are known to influence plant performance, providing a conceptual basis for evaluating their effects on grapevine growth. Based on their complementary PGPT profiles and demonstrated compatibility, these strains were assembled into four bacterial consortia (BC1–BC4). The consortia were applied by root dipping and soil inoculation to one-year-old grapevines subjected to progressive drought stress, in order to assess their potential synergistic effects on plant growth. Treated vines were compared to a non-inoculated control (NI). Results indicate that while BC1 and BC2 did not significantly impact shoot growth, BC3 and partially also BC4 increased shoot length by 35.5% and 26.5%, respectively, compared to NI. Notably, BC3 enhanced shoot elongation during the early phase under well-watered conditions, whereas BC4 conferred greater benefits under water deficit. After five days of suspended irrigation, BC4-treated vines maintained higher photosynthetic activity and stomatal conductance compared to all other treatments, which, displayed almost complete stomatal closure. This response may be linked to the enrichment of indole-3-acetic acid-producing and exopolysaccharide-forming strains, which are known to modulate plant growth and water status. Finally, both BC3 and BC4 promoted greater root biomass by the end of the pot trial. Overall, the results highlight the potential of rationally tailored PGPR consortia to enhance grapevine growth under both optimal and drought conditions, supporting their application as context-specific MBs for sustainable viticulture.

## 1 Introduction

Plant Growth-Promoting Rhizobacteria (PGPRs) are classified as microbial biostimulant (MBs), constituting a significant subgroup within the broader category of plant biostimulants (du Jardin et al., 2025). These beneficial microorganisms inhabit the rhizosphere, a narrow soil region surrounding plant roots directly influenced by root exudates. Although PGPRs represent only 3-5% of the total rhizobacterial population (Antoun and Kloepper, 2001), they play a critical role in enhancing nutrient acquisition, synthesizing phytohormones, suppressing pathogens and modulate stress-related hormonal pathways (Lucy et al., 2004; Lugtenberg and Kamilova, 2009). Bacterial diversity in the rhizosphere is predominantly represented by three major phyla: Proteobacteria, Firmicutes, and Actinobacteria. Among these, the most extensively studied PGPRs belong to genera such as *Arthrobacter*, *Azospirillum*, *Azotobacter*, *Bacillus*, *Mesorhizobium*, *Pseudomonas*, and *Rhizobium* (Glick, 1995). Beneficial effects have also been observed from strains within genera such as *Enterobacter*, *Erwinia*, *Burkholderia*, *Serratia*, and *Flavobacterium*. Although these genera include species known for opportunistic or pathogenic traits, selected strains have shown promising Plant Growth-Promoting Traits (PGPTs) (Haile et al., 2025; Marghoob et al., 2023; Sanadhya et al., 2025). This variability underscores the need for genomic safety assessment when selecting strains for microbial biostimulant applications in agricultural systems. Whole-Genome Sequencing (WGS) enables the evaluation of their functional potential while identifying undesirable elements such as antimicrobial resistance (AMR) genes, virulence factors (VF) and mobile genetic elements that may spread through soil communities (Bellotti et al., 2025; Riva et al., 2024).

Despite advances in PGPR isolation and characterization, the performance of single-strain inoculants often remains inconsistent under variable environmental conditions (Finkel et al., 2017). Microbial consortia are therefore gaining attention, as complementary functional traits and synergistic interactions can enhance and stabilize plant responses (Liu et al., 2023). Yet incompatibility among strains or reduced plant performances compared to individual inoculant has also been reported (Paredes et al., 2018; Rolli et al., 2015), supporting the need to evaluate microbe–microbe interactions prior to formulation under realistic, field-like conditions (Tabassum et al., 2017). For grapevine, however, functionally validated and biosafe PGPR consortia specifically designed to improve drought resilience remain scarce, representing a relevant knowledge gap.

This gap is particularly relevant for viticulture, one of the most important perennial crops worldwide, not only for its extensive cultivation but also for the economic and cultural value of wine production. The sector is increasingly challenged by climate change, as rising temperatures, altered precipitation patterns and more frequent extreme events intensify drought stress. The impact is especially severe in semi-arid regions such as the Mediterranean and in rainfed vineyards, where water scarcity already limits shoot and root development in young vines, compromising establishment and delaying the onset of production (Santillán et al., 2020; Gutiérrez-Gamboa et al., 2021; Palliotti et al., 2014). Consequently, enhancing vineyard resilience to drought has become a key priority for both growers and researchers.

Some studies have explored the potential of microbial inoculants to improve grapevine tolerance to abiotic stresses (Dagher et al., 2025), although relatively few have specifically addressed drought. For instance, Duan et al. (2021) demonstrated that mixed inoculation of drought-tolerant rhizobacteria improved nutrient uptake, hormonal balance, and antioxidant capacity in one-year-old grapevines. While encouraging results, current evidence remains limited and insufficient to guide the development of robust, multi-strains PGPR consortia tailored to grapevine under water-limited conditions.

Rhizosphere microbial communities often reflect the physiological status of the host plant. Under drought conditions, grapevines may selectively recruit microorganisms with traits that enhance stress tolerance, consistent with the “cry-for-help” concept, whereby specific root exudates promote the enrichment of beneficial taxa (Cesco et al., 2021; Rolfe et al., 2019). Such drought-associated microbes represent valuable candidates for microbial biostimulant (MB) development, as they may already carry functional traits aligned with resilience to water limitation (de Vries et al., 2020; Vurukonda et al., 2016). Selective media can facilitate the isolation of beneficial rhizobacteria, while high-throughput *in vitro* assays enable efficient PGPT screening (Guerrieri et al., 2020). Matching strain origin to the environmental conditions of the application site has also been proposed as an effective strategy to improve inoculant performance (Compant et al., 2019; Finkel et al., 2020).

Based on these premises, we developed a comprehensive experimental workflow combining isolation, *in vitro* screening, genomic characterization, and *in vivo* evaluation of drought-tolerant PGPR associated with grapevine roots.

The specific aims of this study were to: i) isolate and identify drought-tolerant PGPRs from the rhizosphere of drought-stressed grapevines; ii) characterize and rank these isolates based on key PGPTs relevant to nutrient mobilization, drought resilience and genomic biosafety; and (iii) evaluate the potential of bacterial consortia (BC), assembled from top-ranked PGPR, to enhance the growth of grapevines under experimentally induced progressive drought stress.

## 2 Materials and methods

### 2.1 Isolation of cultivable rhizobacteria

Rhizosphere soil samples were collected in July 2023 from three different *Vitis vinifera* L. sources: i) a 20-year-old, non-irrigated vineyard of cv. Barbera grafted to 420A rootstock, located in the Colli Piacentini area (44°57′ N, 9°42′ E); ii) 3-year-old potted vines of cv. Sangiovese grafted to SO_4_ rootstock and iii) a 6-year-old experimental vineyard of cv. Ortrugo grafted to SO_4_ rootstock at Università Cattolica del Sacro Cuore in Piacenza (45°02′ N, 9°43′ E). Site i) was selected as a typical non-irrigated hillside vineyard (with an annual rainfall of 724.4 mm in 2023), with low organic matter content (1.5% in the 0-30-cm layer) and a spontaneous, permanent inter-row cover crop, where vines typically experience severe stress during the summer. Site ii) was included for the possibility to control irrigation, soil water and plant water status at the time of sampling. Site iii) represents an irrigated vineyard in the Po Valley and was included to enable control over irrigation and plant water status at the time of sampling, as well as to encompass a third local cultivar. At the time of sampling, mowed spontaneous grass cover was present under the vine row in both the Barbera and Ortrugo vineyards, while bare soil was maintained in the Sangiovese pots.

Rhizosphere samples were collected from the three above-mentioned sites between 12:00 p.m. and 1:00 p.m., when midday leaf water potentials (Ψ_MD_) were between −1.3 and −1.4 MPa, values typically associated with water deficit in grapevine (Deloire et al., 2020). Soil sampling involved extracting two soil cores per vine from three selected grapevines, at a depth of 25–30 cm and approximately 20 cm from the vine trunk. The cores were placed in sterile polybags and kept in a cooler with ice packs during transport to the laboratory.

Rhizosphere soil was recovered by washing root fragments from each soil core with sterile 0.9% saline solution supplemented with Tween 80 (0.01% v/v), following Barillot et al. (2013). Rhizosphere soil (10 g) was suspended in sterile saline solution, homogenized under agitation, serially diluted up to 10⁻^7^, and plated in triplicate on selective and non-selective agar media according to expected bacterial abundance. The following rich or semi-selective media were used: TSA (Tryptic Soy Agar, Oxoid, Basingstoke, UK), SCNA (Starch-Casein Nutrient Agar) for *Streptomyces* spp., and PSB (Pseudomonas Agar Base, Oxoid, Basingstoke, UK) for *Pseudomonas* spp. were employed. Moreover, to select spore-forming *Bacillus* spp., soil suspensions (10^-1^) were agitated for 90 mins in 55-60% ethanol and then plated on TSA. To prevent fungal growth, all media were amended with cycloheximide (0.1%, Sigma-Aldrich, St. Louis, MO, USA) and incubated at 30°C for 24–72 hours.

For the enrichment of drought resistance rhizobacteria, three liquid media TSB (Tryptic Soy Broth, Oxoid, Basingstoke, UK), NFb (Nitrogen-free broth), and MEB (Malt Extract Broth, Oxoid, Basingstoke, UK) were supplemented with 24% (w/v) PEG 6000 (polyethylene glycol 6000, Merck, Germany). After three consecutive enrichment steps at 30°C in shaking at 180 rpm, bacterial suspensions were serially diluted and plated in triplicate on TSA, then incubated at 30°C for 24–72 h. Morphologically distinct colonies were isolated, and axenic cultures were cryopreserved at -20°C in 20% (v/v) glycerol. Strain IDs were initially assigned based on the cultivar of origin: Barbera (BA), Sangiovese (SG), and Ortrugo (OR).

### 2.2 Bacterial molecular genotyping

Genomic DNA was extracted from pure cultures of rhizobacterial isolates using the MicroLYSIS^®^ Plus Kit (Microzone, Haywards Heath, UK). Genotyping fingerprinting was used to identify duplicate strains and was performed using the rep-PCR technique, as described by Sadowsky and Hur (1998). In brief, the primer (GTG)5 (5′-GTGGTGGTGGTGGTG-3′) was used for the amplification of repetitive elements, and PCR products were separated on a 2.5% agarose gel stained with SYBRsafe. Digital images of gel banding patterns were analyzed using GelJ v2.0 software to identify genetically unique isolates. Duplicate strains (i.e., isolates showing identical rep-PCR patterns) were excluded from further analysis.

Taxonomic identification of the remaining unique strains was performed through amplification of the 16S rRNA gene using the universal primer pair 27F (5′-AGAGTTTGATCCTGGCTCAG-3′) and 1492R (5′-GGTTACCTTGTTACGACTT-3′), according to the protocol described by Cello and Fani (1996). The PCR reaction mixture and cycling conditions are described in Guerrieri et al. (2020). Amplified PCR products were purified using the ReliaPrep™ PCR Purification Kit (Promega, Madison, WI, USA) and submitted for Sanger sequencing at Eurofins Genomics (Cologne, Germany). The resulting sequences were queried against the NCBI nucleotide database using the BLASTN algorithm (https://blast.ncbi.nlm.nih.gov/Blast.cgi). Taxonomic assignment was based on the best hit, with a species-level identity threshold set at >98.7%.

### 2.3 Inoculation and screening of *in vitro* PGPTs

All phenotypic assays were performed following the protocol of Bellotti et al. (2023). Briefly, strains were cultured on TSA and transferred to TSB for overnight growth at 30°C. Standardized bacterial suspensions (3 x 10^8^ CFU/ml) were then inoculated onto assay-specific media for PGPT quantification.

Due to the high number of isolates (n=124), drought tolerance was used as the first selection criterion, and only isolates performing well under PEG-induced osmotic stress were selected for further PGPT screening. All assays were carried out in triplicate.

#### 2.3.1 *In vitro* drought tolerance assay

The relationship between PEG concentration and water potential (Ψ_w_) was assessed in TSB medium amended with increasing concentrations of PEG 6000 (0%, 12%, 24%, and 32%), maintained at 30 °C. Ψ_w_ was measured after 24 and 48 hours using a WP4C PotentiaMeter (METER Group, Inc., USA), via the chilled-mirror dew-point technique, which measures both osmotic and matric components, with a range from 0 to 300 MPa and high accuracy. All measurements were performed in precise mode using stainless steel sample cups. This allowed us to establish a direct relationship between PEG concentration and the actual water potential of the medium, ensuring a precise quantification of drought-like stress levels. These PEG concentrations were selected because they reproduce water potentials ranging from moderate drought to the soil permanent wilting point (Ψ_w_ = -1.5 MPa), allowing us to evaluate bacterial growth across a physiologically relevant drought gradient.

For the drought tolerance screening, osmotic stress was imposed to simulate drought-like conditions, and 124 bacterial isolates were inoculated into 300 µL of TSB medium supplemented with PEG 6000 (0%, 12%, 24%, and 32%, corresponding to Ψ_w_ values of -0.72, -0.98, -1.67, and -2.26 MPa, respectively) in 96-well plates. Growth was monitored by optical density at 600 nm (OD_600_) readings at 12, 24 and 48 h using a Synergy microplate reader (BioTek). Based on OD_600_ values at 48 h, strains were classified as: highly sensitive (<0.3), sensitive (0.3-0.4), tolerant (0.4-0.5), or highly tolerant (>0.5), following Susilowati et al. (2018). Only highly tolerant strains at 24% PEG (Ψ_w_ ≈ -1.67 MPa) were retained for downstream PGPT assays.

#### 2.3.2 Ammonium formation

Ammonium formation was assessed using a slightly modified plate assay based on the methods described by Abdelwahed et al. (2022) and Tang et al. (2020). 5 µL of bacterial suspension were spot inoculated in triplicate onto NFb agar medium supplemented with 0.1% yeast extract and incubated at 30°C for 24–48 hours. Colony growth and color reactions were used as qualitative indicators of NH_4_^+^ production. A complementary quantitative assay was performed in peptone water broth, where ammonia release was detected using Nessler’s reagent and measured spectrophotometrically (Thermo Scientific Multiskan EX, Waltham, Massachusetts, USA) at 450 nm. The NH_3_ concentration was estimated with a standard curve of (NH_4_)_2_SO_4_ solution in peptone water.

#### 2.3.3 Phosphorous solubilization

Phosphorus solubilization (PS) screening was performed using NBRIP agar and broth media supplemented with 0.025 mg/mL bromophenol blue (BPB) and referred to as NBRIP-BPB (Nautiyal, 1999). 5 µL of bacterial suspensions were spotted in triplicate onto NBRIP agar plates and 5 µL inoculated into NBRIP-BPB broth, followed by incubation at 30°C for 7 days.

On NBRIP agar, the phosphate solubilization index (PSI) was determined by measuring the diameter of the colony and the surrounding halo. PSI was calculated as:


$$PSI\%=\;\frac{Ø\;(colony+halo)\;}{Ø\;(colony)}\times 100$$

In NBRIP-BPB broth, PS activity was quantified spectrophotometrically based on the color shift associated with medium acidification. Results were expressed as percent phosphorus units (PPU), using the same formula adopted for siderophore production.

#### 2.3.4 Potassium solubilization

Potassium solubilization (KS) screening was assessed using Aleksandrov agar medium (Alek) (Rajawat et al., 2016) supplemented with 0.025 mg/mL of bromophenol blue (BPB). 5 µL of bacterial suspension was spotted in triplicate onto Alek-BPB. After 5 days of incubation at 30°C, potassium solubilization was evaluated based on the yellow halo formed around the colonies. Results were expressed as Potassium Solubilization Index (KSI), determined by measuring the diameters of the colonies and the corresponding clear zones, where:


$$KSI\%=\;\frac{Ø\;(colony+halo)\;}{Ø\;(colony)}\times 100$$

#### 2.3.5 Siderophores production

Siderophore (SID) production was evaluated using the Siderophore Inducing Medium added with Chrome Azurol Sulphonate (SIM-CAS) assay, following the method of Schwyn and Neilands (1987) as described by Dimkpa (2016). 5 µL of bacterial suspension were inoculated in SIM medium and incubated at 30°C for 7 days. Culture supernatants were reacted with CAS reagent to quantify the color shift from blue to orange/yellow, which indicates siderophore production. Siderophore production was expressed as percent siderophore units (PSU), calculated as:


$$PSU\%=\;\left[\frac{\;\left(Ar\;-\;As\right)\;}{Ar}\right]\times 100$$

where *Ar* is the absorbance of the reference (CAS reagent + uninoculated medium) and *As* is the absorbance of the sample.

#### 2.3.6 Indole-3-acetic acid production

Auxin hormone production was quantified using Salkowski colorimetric assay following the protocol of Harikrishnan et al. (2014). 5 µL of bacterial suspension were inoculated in LB medium supplemented with 0.1% L-tryptophan (Sigma-Aldrich, St. Louis, MO, USA) and incubated at 30°C for 7 days. After incubation, cultures were centrifuged and the supernatant was reacted with Salkowski reagent. IAA concentration (µg/mL) was determined using a standard curve. The nine isolates selected for the pot experiment were further tested for IAA production simulating water deficiency condition, inoculating the strains in LB medium amended with 18% PEG 6000.

#### 2.3.7 Exopolysaccharides production

The screening of Exopolysaccharides (EPS) production was performed following the protocol described by Nwosu et al. (2019), with minor modifications. Briefly, 1 mL of bacterial cell suspensions were inoculated into 50 mL of TSB supplemented with 2% sucrose and incubated on a rotary shaker at 30°C for 72 hours at 120 rpm. After incubation, cultures were centrifuged and EPS was precipitated from the supernatant using chilled ethanol. The precipitate was recovered by centrifugation, dried at 60°C to constant weight, and quantified gravimetrically.

The nine isolates selected for the pot experiment were further tested for EPS production in TSB supplemented with 2% sucrose and 18% PEG 6000.

#### 2.3.8 Biofilm formation

Biofilm formation was assessed using the Congo Red Agar (CRA) method described by (Freeman et al., 1989). Strains were plated on CRA medium and incubated at 37°C for 24–48 h. Colonies displaying black, dry, crystalline morphology were classified as biofilm producers, whereas all other morphologies were considered non-producers.

### 2.4 Ranking system and compatibility testing

To select the most promising bacterial isolates, a ranking system was developed based on the cumulative performance across all *in vitro* PGPT assays. A modified version of the scoring system proposed by (Guerrieri et al., 2020) was applied. For each PGPT, the full range of observed values was divided into five equal intervals, corresponding to performance levels from 1 (lowest) to 5 (highest). These levels were converted into a ratio scale: Level 1 = 0, Level 2 = 0.25, Level 3 = 0.5, Level 4 = 0.75, and Level 5 = 1.

For qualitative traits such as biofilm formation, a binary scoring system was adopted, using values of 1 and 0 assigned to positive and negative results, respectively. Moreover, the growth performance of drought-tolerant isolates in TSB supplemented with 24% PEG 6000 (OD_600_ ranging from 0.51 to 1.00) was divided into five levels and taken into account in the ranking. The scores obtained for each trait were then summed for each isolate to calculate the final performance ranking, which was further categorized into nutritional and drought-tolerance features. The nine top-ranked strains, excluding potential pathogens, were selected to assemble the BC used in the subsequent pot experiment. BC were assembled based on three main criteria: (i) complementarity in PGPT expression, (ii) potential synergistic effects based on shared cultivar origin, and (iii) absence of incompatibility among strains.

To assess inter-strain compatibility, an *in vitro* plate-based interaction assay was conducted, following the protocol of Wang et al. (2024). Briefly, overnight cultures (OD_600_ = 1.0) were diluted to 1:100 and incorporated into molten TSA maintained at approximately 30°C. The inoculated agar was then poured into sterile Petri dishes (20 mL per plate) and allowed to solidify, resulting in a uniform distribution of the background strain within the medium. Once solid, 5 μL of each of the remaining candidate strains were spot-inoculated onto the surface of the plate in equidistant positions. Plates were incubated at 30°C for 48 hours and examined for growth inhibition. The presence of a clear inhibition halo surrounding a spot indicated antagonistic interaction, while its absence was interpreted as compatibility. Strains that exhibited antagonism were excluded from consortia and replaced with compatible alternatives. All compatibility assays were performed in triplicate for each pairwise combination.

### 2.5 Whole-genome sequencing of selected strains and screening for phenotypic traits, antibiotic resistance genes, virulence factors, and toxins

WGS and *in silico* analyses were performed on the nine bacterial strains selected for the pot experiment to investigate their full potential for plant growth promotion and assess biosafety-related features.

Genomic DNA (gDNA) was extracted from TSB overnight cell cultures in exponential phase using the E.Z.N.A.^®^ Bacterial DNA Kit (Omega Bio-tek, Norcross, GA, USA), following the manufacturer’s instructions. gDNA quality was assessed by electrophoresis on a 1% (w/v) agarose gel to verify integrity and quantified using the Qubit™ 4 Fluorometer with the dsDNA HS Assay Kit (Thermo Fisher Scientific, USA). High-quality gDNA was sequenced at Novogene (Cambridge, UK) using the Illumina NovaSeq platform, generating 250 bp paired-end reads.

Genome assembly was performed using the BV-BRC pipeline (https://www.bv-brc.org/) which employs Unicycler v0.5.1 as the default assembler strategy for short reads. Assembled genomes were annotated using the RAST tool kit (RASTtk) integrated into the BV-BRC platform (Olson et al., 2023). Taxonomic classification at the genome level was conducted using the Similar Genome Finder function on BV-BRC, which compares query genomes against a curated set of high-quality reference genomes using a Mash/MinHash-based distance algorithm. For each strain, the closest match was selected based on the highest similarity score, providing species-level resolution and validation of the 16S rRNA gene-based classification, especially in cases of closely related taxa or ambiguous assignments.

To assess biosafety, predicted contigs were screened for the presence of AMRs genes and VFs using ABRicate v1.0.0 (https://github.com/tseemann/abricate). AMR gene detection was performed using the ResFinder and CARD databases (Alcock et al., 2020; Bortolaia et al., 2020), while virulence determinants were identified using the VFDB (Chen et al., 2005). Only hits showing ≥80% sequence identity and ≥70% alignment coverage were retained, in accordance with EFSA guidelines for microbial genome screening (EFSA et al., 2024).

In addition, predicted protein-coding sequences were uploaded to the PGPT-Pred tool in PLaBAse (Patz et al., 2021) to annotate genomic traits associated with plant growth promotion. The resulting profiles provided functional insight into genes involved in nutrient solubilization, hormone production, stress tolerance, and root colonization traits.

### 2.6 Preparation of consortia and application on vines

#### 2.6.1 Preparation of plant inoculants

Each of the nine selected bacterial strains was individually cultured by transferring a single colony into a 10 mL tube containing 5 mL of TSB medium, incubated overnight at 30°C. Cultures were then standardized to 3 × 10^8^ CFU/mL, and 5 µL were inoculated into 25 mL of TSB in 100 mL Erlenmeyer flasks and incubated for 24 h (30°C, 145 rpm). After incubation, cell densities were determined by CFU counting. Standardizing the initial inoculum allowed us to compare growth kinetics across strains and to define the target cell concentration used for the preparation of the final inocula.

Depending on the strain’s growth rate, between 0.1 and 100 µL were inoculated into 5000 mL Erlenmeyer flasks containing 2000 mL of medium for *Bacillus* spp. and into 3000 mL Erlenmeyer flasks containing 1700 mL of medium for all other genera, followed by incubation in a rotary shaker (145 rpm, 30°C) for 24 hours.

Cultures were harvested by centrifugation at 5000×g for 10 min at room temperature using sterile 250 mL centrifuge bottles. The resulting cell pellets were resuspended in sterile physiological saline solution (0.9% NaCl, w/v). Equal volumes of the individual bacterial suspensions were then combined to assemble four microbial consortia. Before application, each consortium was diluted with tap water to a final concentration of 10^8^ CFU/mL.

#### 2.6.2 Plant material, experimental setup, water supply and inoculation

The trial, conducted in 2024 at Università Cattolica del Sacro Cuore in Piacenza, Italy (45°02’N, 9°43’E), tested the nine most promising strains assembled into different consortia. The experiment used 30 one-year-old grapevines (*Vitis vinifera* L. cv. Sangiovese, clone R24 grafted onto SO4 rootstock), grown outdoors in 14 L pots filled with a non-sterilized 70:30 sand-to-soil mixture. The substrate consisted of 70.7% sand, 17.3% silt and 12.0% clay, with pH 8.2, total nitrogen 0.45 g kg⁻¹, assimilable phosphorus 66.9 mg kg⁻¹, exchangeable potassium 0.24 cmol^+^ per 100 g, cation-exchange capacity 14.4 meq per 100 g, and organic matter 0.69**%**. Once vines reached 3–4 unfolded leaves (BBCH 13 stage, according to Lorenz et al. (1995), were thinned to two shoots per plant. The cultivar and the rootstock were selected due to their widespread use in Italian viticulture.

Plants were randomly positioned in three rows of ten pots. Treatments were then assigned in a systematic repeated sequence (NI, BC1, BC2, BC3, BC4), ensuring balanced replication and an even distribution of treatments across the rows. This resulted in six biological replicates per treatment. The consortia were assembled from the nine strains identified as most promising through a stepwise selection workflow that included: (i) isolation from drought-exposed grapevine rhizospheres, (ii) *in vitro* screening for drought tolerance and key PGPTs, and (iii) ranking based on overall functional performance. These top-ranking strains were then combined into consortia according to criteria of complementarity in PGPT expression, potential synergistic interactions related to their shared cultivar origin, and the absence of incompatibility. Root inoculation was performed at planting (March 27^th^) by soaking trimmed roots in 4 L of the corresponding microbial consortium suspension (10^8^ CFU/mL) for 6 h. A second soil inoculation was carried out on April 29th, when vines had reached the BBCH 16 stage (5–6 unfolded leaves), by applying 100 mL of the same suspension directly to the soil. Control vines received equivalent treatments using tap water. After inoculation, each pot was covered with a plastic bag to prevent rainfall-driven leaching of the inoculum. No fertilization was applied throughout the experiment.

Daily evapotranspiration (ET) was estimated once per week using a gravimetric method: three additional well-watered vines (at field capacity after draining excess water) were weighed at 8:00 a.m. and reweighed 24 hours later; the difference in weight was used as the daily ET. All the experimental vines were watered daily at 100% ET until June 19^th^, when plants had developed more than 10 unfolded leaves. From this date, water stress was gradually imposed by reducing irrigation from 100% to 60% ET (for 9 days), and then to 0% (for 6 days), to simulate a progressive decline in soil water availability. The dry-down period lasted until July 3rd, when most vines exhibited stomatal closure.

#### 2.6.3 Vegetative parameters, physiological measurements, and biomass quantification

Shoot length was recorded weekly on each vine, starting from the second inoculation day.

During the dry-down phase, physiological measurements were carried out on four dates: i) June 19^th^, when all vines were fully watered (100% ET); ii) June 22^nd^, with irrigation at 60% ET; iii) June 29^th^ and July 2^nd^, corresponding to the 2^nd^ and 5^th^ day after irrigation withdrawal.

The measurements included leaf gas exchange and leaf water potential, performed on one fully expanded, mid-shoot leaf per plant (three replicates per treatment). Gas exchange, including photosynthetic rate (A), stomatal conductance (g_s_), and transpiration rate (E), was measured at midday under saturating light conditions (PPFD > 1200 µmol m^-^² s^-^¹) using a portable photosynthesis system (LCi-SD, ADC Bioscientific Ltd., Hoddesdon, UK) equipped with a 6.25 cm² broad-leaf cuvette and a constant airflow rate of 200 mL min^-^¹. Midday leaf water potential (Ψ_MD_) was then measured on the same leaves using a Scholander pressure chamber (Model 3500, Soilmoisture Equipment Corp., Santa Barbara, CA). Predawn leaf water potential (Ψ_PD_) was measured at 04:00 on the same days, on the same plants, using the same instrument.

At final harvest, plants were destructively separated into shoots, leaves, trunk, and roots. Fresh biomass was recorded for each organ, and total leaf area per vine was estimated by multiplying the number of nodes by the average leaf area of both principal and lateral leaves, which was calculated using a leaf area meter (LI-3100C, LI-COR, Lincoln, NE, USA) on samples from six randomly selected vines. All tissues were then oven-dried at 105 °C until constant weight to determine dry biomass and organ-specific allocation.

### 2.7 Statistical analyses

All data analyses for the *in vitro* phenotypic assays and grapevine performance parameters were performed using one-way analysis of variance (ANOVA) in RStudio (v4.3.1). When significant effects were detected (*p*< 0.05), *post hoc* comparisons between treatment means were conducted using the Fisher’s Least Significant Difference (LSD) test. Graphical representations of the data were generated using the ggplot2 package in R.

## 3 Results

### 3.1 Isolation, identification, and characterization of drought-tolerant rhizobacteria

A total of 132 rhizobacterial isolates were obtained from the rhizosphere of *V. vinifera* cultivars Barbera, Ortrugo, and Sangiovese grown under water deficit (-1.5 MPa ≤ Ψ_MD_ ≤ -1.3 MPa). Genotypic fingerprinting by rep-PCR identified eight duplicate isolates with identical banding patterns (data not shown), which were excluded from further analyses. The remaining 124 unique isolates (**Supplementary Table S1**) were used for downstream analysis. At the Phylum level, the isolates belonged to *Firmicutes* (54.39%), *Proteobacteria* (28.07%), *Actinobacteria* (14.91%), and *Bacteroidota* (2.63%).

To prioritize isolates for functional assays, a pre-screening under osmotic stress (24% PEG 6000, Ψw = -1.67 MPa) was used to select drought-tolerant candidates. A total of 50 isolates exhibited robust growth (OD_600_ > 0.5) after 48 hours of incubation and were selected for *in vitro* phenotypic tests (**Table 1**). According to the 16S rDNA sequencing, the 50 drought-tolerant isolates (21 strains from cv. Sangiovese, 17 from cv. Ortrugo, and 12 from cv. Barbera) were taxonomically assigned to 22 different genera. The most frequently represented genera were *Pseudomonas* (24.5%), *Streptomyces* (20.4%), *Bacillus* (10.2%), *Priestia* (6.1%), and *Cupriavidus* (4%). *Streptomyces* and *Pseudomonas* exhibited the greatest species richness, with 10 and 9 unique species, respectively, followed by *Bacillus* with 4 species. Several additional genera commonly associated with PGP activity were also identified, each represented by a single isolate, including *Enterobacter ludwigii*, *Paenibacillus susongensis*, *Variovorax paradoxus*, *Serratia rhizosphaerae*, and *Sinorhizobium arboris*.

**Table 1:** Functional ranking of the 50 best drought-stress-tolerant rhizobacteria isolates based on their *in vitro* PGPTs, categorized into nutritional (N traits) and drought-related (D traits).

Code	Taxonomy	NH_4_^+^	KS	PS-plate	PS-broth	SID	NH_3_	EPS	BF	IAA	DT	Rank		
												N trait	D trait	Tot
UC4449*	*Pseudomonas glycinis*	1	1	1	1	0.75	0.5	1	1	0.5	0.5	5.25	3	8.25
UC4489	*Pseudomonas aeruginosa*	1	1	0.75	1	1	0.5	0.75	0	0	1	5.25	1.75	7
UC4450*	*Enterobacter ludwigi*	1	0.75	0.75	0.5	1	1	0	0	1	0.75	5	1.75	6.75
UC4521*	*Bacillus licheniformis*	0.75	1	0	0.25	1	1	1	1	0	0.5	4	2.5	6.5
UC4441	*Pantoea stewartii*	1	1	0.5	1	0.75	0	0.75	1	0.25	0.25	4.25	2.25	6.5
UC4439*	*Pseudomonas frederiksbergensis*	1	0.5	1	0.75	0.75	0.5	1	1	0	0.25	4.25	2.25	6.5
UC4553*	*Pseudomonas plecoglossicida*	1	1	0.5	0.75	1	0.25	0.25	0	0	1	4.5	1.25	5.75
UC4444	*Bacillus velezensis*	1	0	0.75	0.5	0.5	0.5	1	1	0.25	0.25	3.25	2.5	5.75
UC4478*	*Pseudomonas chlororaphis*	1	0	0.5	0.25	0.75	0.5	1	0	0.5	1	3	2.5	5.5
UC4510*	*Priestia megaterium*	1	0.25	0	0.75	1	0	0.75	1	0.5	0.5	3	2.25	5.5
UC4490*	*Serratia rhizosphaerae*	1	0.75	0.75	1	0	0.25	0.5	0	0	1	3.75	1.5	5.25
UC4535*	*Pseudomonas furukawaii*	1	0	0	0.25	1	0.5	0.25	0	0.25	1	2.75	2.5	5.25
UC4549	*Pseudomonas furukawaii*	1	0	0.75	0	0.75	0.25	0.25	1	0	0.75	2.75	2	4.75
UC4479	*Pseudomonas lini*	1	0.75	0.5	0.75	0.5	0.25	1	0	0	0	3.75	1	4.75
UC4443	*Bacillus amyloliquefaciens*	1	0	0.75	0	0.25	0.25	1	1	0	0.25	2.25	2.25	4.5
UC4442	*Cupriavidus necator*	1	0	0.75	0.25	0	0.25	0.5	1	0	0.25	2.25	1.75	4
UC4446	*Bacillus amyloliquefaciens*	1	0.25	0	0.5	0.5	0.5	1	0	0	0.25	2.75	1.25	4
UC4481	*Acinetobacter couvalinii*	1	0.75	0.75	0.25	0.25	0.25	0.25	0	0	0.25	3.25	0.5	3.75
UC4440	*Bacillus atrophaeus*	1	1	0.5	0.25	0	0	0.75	0	0	0.25	2.75	1	3.75
UC4445	*Kokuria rosea*	1	0	0	0.25	1	0.25		1	0	0.25	2.5	1.25	3.75
UC4526	*Pseudomonas hibiscicola*	1	0	0	0	0.75	0.5	0.5	0	0.25	0.5	2.25	1.25	3.5
UC4527	*Pseudomonas furukawaii*	1	0.5	0	0.25	0.25	0.5	0.25	0	0	0.75	2.5	1	3.5
UC4545	*Priestia megaterium*	0.75	0.25	0	1	0.5	0.25	0.25	0	0	0.5	2.75	0.75	3.5
UC4546	*Priestia megaterium*	0	0.25	0	1	0.25	0.5	0.25	1	0	0.25	2	1.5	3.5
UC4460	*Streptomyces albireticuli*	0	0	0	0	0.25	0.25	1	1	0.5	0.5	0.5	3	3.5
UC4495	*Krasilnikoviella muralis*	0.75	0	0.5	0.25	0	0	0.75	1	0	0.25	1.5	2	3.5
UC4551	*Pseudomonas boreopolis*	1	1	0	0.5	0	0	0.5	0	0	0.25	2.5	0.75	3.25
UC4484	*Calidifontibacillus erzurumensis*	0.5	0	0	0.5	0.5	0	1	0	0	0.75	1.5	1.75	3.25
UC4498	*Peribacillus simplex*	0.75	0	0.75	0.25	0	0	0.75	0	0	0.75	1.75	1.5	3.25
UC4533	*Microbacterium foliorum*	1	0.25	0	0	0	0.25	0.75	0	0	0.75	1.5	1.5	3
UC4485	*Empedobacter brevis*	0	0	0.5	0	0	0	1	1	0.25	0.25	0.5	2.5	3
UC4491	*Sphingobacterium pakistanense*	0	0	0.5	0.5	0	0.25	0.5	1	0	0.25	1.25	1.75	3
UC4534	*Pseudomonas furukawaii*	1	0	0	0.25	0.25	0.5	0.5	0	0	0.25	2	0.75	2.75
UC4461	*Variovorax paradoxus*	1	0	0.75	0	0	0.25	0	0	0	0.75	2	0.75	2.75
UC4467	*Paenibacillus susongensis*	0.5	0	0.5	0	0	0.25	0	1	0	0.5	1.25	1.5	2.75
UC4509	*Sinorhizobium arboris*	1	0.25	0	0	0	0.5	0	0	0.75	0	1.75	0.75	2.5
UC4454	*Streptomyces canus*	0	0	0	0	0	0.25	0.5	0	1	0.5	0.25	2	2.25
UC4492	*Streptomyces violaceochromogenes*	0	0	0	0	0	0.25	0.5	1	0	0.5	0.25	2	2.25
UC4497	*Streptomyces endophyticus*	0.75	0	0	0.25	0	0	0.5	0	0	0.5	1	1	2
UC4507	*Rossellomorea marisflavi*	0.5	0	0	0.25	0	0.25	0	0	0.25	0.75	1	1	2
UC4516	*Bacillus zanthoxyli*	0	0	0	0.5	0.25	0	0	0	0.5	0.75	0.75	1.25	2
UC4437	*Streptomyces mauvecolor*	0	0	0	0	0	0.25	1	0	0.25	0.5	0.25	1.75	2
UC4548	*Rhizobium wuzhouense*	0	0	0	0	0	0.25	0.25	1	0	0.25	0.25	1.5	1.75
UC4493	*Streptomyces chartreusis*	0	0	0	0.25	0	0.25	0.75	0	0	0.5	0.5	1.25	1.75
UC4501	*Cupriavidus cauae*	1	0	0	0	0	0	0.25	0	0	0.25	1	0.5	1.5
UC4554	*Streptomyces arenae*	0	0	0	0	0	0.25	0.75	0	0	0.5	0.25	1.25	1.5
UC4556	*Streptomyces albogriseolus*	0	0	0	0	0	0.25	0.75	0	0	0.5	0.25	1.25	1.5
UC4557	*Streptomyces collinus*	0	0	0	0	0	0.25	0.5	0	0	0.5	0.25	1	1.25
UC4434	*Streptomyces mauveocolor*	0	0	0	0	0	0	0.75	0	0	0.5	0	1.25	1.25
UC4511	*Brevibacillus laterosporus*	0	0	0	0	0	0	0.5	0	0	0.5	0	1	1

The nine top performing strains selected for the pot trials are indicated with an asterisk (*). NH_4_^+^/NH_3_, Ammonium/Ammonia production; KS, Potassium solubilization; PS-plate, Phosphorous Solubilization in agar; PS-broth, Phosphorous Solubilization in broth; SID, Siderophore production; EPS, Exopolysaccharides production; BF, Biofilm formation; IAA, Indole-3- acetic acid production; DT, drought tolerance; assessed based on OD_600_ values ranging from 0.5 to 1 at -1.67 MPa (PEG 6000 at 24%). Values represent PGPTs scores scaled from 1 to 5 and converted to a 0–1 ratio (0, 0.25, 0.5, 0.75, 1).

To evaluate and compare the functional potential of the 50 drought-tolerant strains, a standardized ranking method was applied based on *in vitro* expression of PGPTs. Each of the 50 strains exhibited at least one detectable PGPT *in vitro*. The complete list of isolates ranked according to the performance in each phenotypic assays is reported in **Table 1**, while the corresponding PGPT quantitative (mean ± standard deviation) or qualitative values are provided in **Supplementary Table S2**.

### 3.2 Drought stress-tolerant PGPR selection and consortia design for pot experiment

Based on the results of the PGPT ranking scores and phenotypic performance, nine PGPR strains were selected for the further *in planta* study. Selection was based on either i) top overall ranking, ii) strong performance under simulated drought conditions (PEG 6000), or iii) the presence of specific traits relevant to either inducing drought resilience or providing nutritional support. Three strains that performed well in the overall ranking were not selected due to either pathogenicity clearly associated with the species namely *P. aeruginosa* UC4489 (2^nd^), *Pantoea stewartii* UC4441 (5^th^) or slow growing strains like *B. velezensis* UC4444 (8^th^), characteristics that would make them unsuitable for industrial upscale and field applications.

The final nine strains selected were: *P. glycinis* UC4449, *E. ludwigii* UC4450, *B. licheniformis* UC4521, *P. frederiksbergensis* UC4439, *P. plecoglossicida* UC4553, *P. chlororaphis* UC4478, *P. megaterium* UC4510, *S. rhizosphaerae* UC4490, and *P. furukawaii* UC4535. To further investigate their potential as MBs, these strains were subsequently tested for IAA and EPS production under osmotic stress induced by 18% PEG 6000 (Ψw = -1.25 MPa). **Table 2** gives a complete overview of the PGPT profiles of the nine selected strains. Notably, among the drought traits, all isolates showed increased EPS production under PEG-induced osmotic stress, with *P. plecoglossicida* UC4553 and *E. ludwigii* UC4450 showing the strongest responses (77× and 499× fold change, respectively). IAA production under optimal conditions, ranged from 8.38 to 46.51 µg/mL. The highest values were observed in *E. ludwigii* UC4450 (46.51 µg/mL), *P. glycinis* UC4449 (24.75 µg/mL), *P. chlororaphis* UC4478 (17.40 µg/mL), and *B. licheniformis* UC4521 (16.29 µg/mL). Under PEG-induced osmotic stress, *P. plecoglossicida* UC4553, *P. frederiksbergensis* UC4439, and *B. licheniformis* UC4521 increased IAA production by 88%, 68%, and 51%, respectively. In contrast, *B. licheniformis* UC4521, *E. ludwigii* UC4450, and *P. megaterium* UC4510 showed reductions of 53%, 48%, and 44%, respectively. Despite this reduction, *E. ludwigii* UC4450 maintained relatively high IAA production (24.19 µg/mL), comparable to *P. frederiksbergensis* UC4439, *P. glycinis* UC4450, and *P. chlororaphis* UC4478. Meanwhile, *P. glycinis* UC4449, *S. rhizosphaerae* UC4490, and *P. furukawaii* UC4535 maintained IAA production at levels similar to the control. Biofilm formation was observed in *E. ludwigii* UC4450, *P. chlororaphis* UC4478, and *S. rhizosphaerae* UC4490.

**Table 2:** *In vitro* phenotypic measurements of the 9 selected drought-tolerant rhizobacterial isolates.

Code	Taxonomy	NH_4_^+^	PPU	PSI	SID	NH_3_	KSI	BF	EPS	EPS+18%	IAA	IAA+18%
UC4449	*Pseudomonas glycinis*	+	48.35 ± 0.03	15.5	44.24 ± 0.07	53.11 ± 11.10	37	–	0.23 b ±0.01	3.59 a ±0.14	27.30 ± 1.40	26.0 ± 0.24
UC4450	*Enterobacter ludwigi*	+	35.56 ± 0.04	10	71.43 ± 0.04	102.0 ± 14.74	16	+	0.03 b ±0.01	4.82 a ±0.09	46.51 a ±1.00	24.19 b ±0.61
UC4521	*Bacillus licheniformis*	+	35.59 ± 0.04	32	18.37 ± 0.05	111.2 ± 17.45	32	+	0.35 b ±0.02	1.20 a ±0.23	16.29 a ±1.35	7.64 b ±0.04
UC4439	*Pseudomonas frederiksbergensis*	+	50.65 ± 0.02	19	62.09 ± 0.06	64.86 ± 14.35	14	–	0.18 b ±0.01	3.93 a ±0.95	13.58 b ±1.37	22.82 a ±0.75
UC4553	*Pseudomonas plecoglossicida*	+	48.77 ± 0.03	32	69.26 ± 0.05	44.50 ± 16.52	32	–	0.06 b ±0.01	2.13 a ±0.32	8.59 b ±2.00	16.75 a ±1.42
UC4478	*Pseudomonas chlororaphis*	+	53.18 ± 0.03	7	79.61 ± 0.02	65.88 ± 24.74	7	–	0.72 b ±0.01	4.16 a ±0.36	17.40 b ±0.32	27.25 a ±1.91
UC4510	*Priestia megaterium*	+	47.65 ± 0.04	16	38.09 ± 0.05	16.37 ± 2.30	16	–	0.12 b ±0.01	0.93 a ±0.30	15.42 a ±1.13	8.68 b ±0.42
UC4490	*Serratia rhizosphaerae*	+	65.74 ± 0.02	25	0.93 ± 0.06	34.54 ± 8.56	25	+	0.09 b ±0.01	1.48 a ±0.03	10.56 ± 1.17	11.44 ± 0.48
UC4535	*Pseudomonas furukawaii*	+	32.58 ± 0.07	6	52.27 ± 0.05	52.49 ± 10.32	6	–	0.08 b ±0.04	3.30 a ±0.26	8.38 ± 2.10	11.73 ± 0.87

Phenotypic results are expressed quantitatively as follows: phosphorus solubilization in broth (Percent Phosphorus Unit, PPU, %), phosphorus solubilization on agar (Phosphorus Solubilization Index, PSI), potassium solubilization (Potassium Solubilization Index, KSI), siderophore production (Percent Siderophore Unit, PSU, %), indole acetic acid production without and with 18% PEG (IAA and IAA + 18%, µg/mL), ammonia production (NH_3_, mg/L), and exopolysaccharide production without and with 18% PEG (EPS and EPS + 18%, g/L). Biofilm (BF) and Ammonium (NH_4_^+^) was evaluated qualitatively as presence (+) or absence (-). Data represent the mean values (± SE) of three replicates per isolate. Different letters indicate significant differences in EPS and IAA production between optimal and osmotic stress (+18% PEG, Polyethylene glycol).

Among nutritional traits, P solubilization efficiency (PPU) ranged from 32.58% to 65.74%, with *S. rhizosphaerae* UC4490, *P. chlororaphis* UC4478 and *P. frederiksbergensis* UC4439 being the most efficient. Siderophore production was the highest in *P. chlororaphis* UC4478 (79.61%) *E. ludwigii* UC4450 (71.43%), while most other strains also showed moderate activity (>30%). Ammonia production was particularly elevated in *B. licheniformis* UC4521 (111.2 µg/mL) and *E. ludwigii* UC4450 (102 µg/mL). Potassium solubilization index (KSI) ranged from 6 to 37, with *P. glycinis* UC4449 ranking the highest.

**Figure 1:**
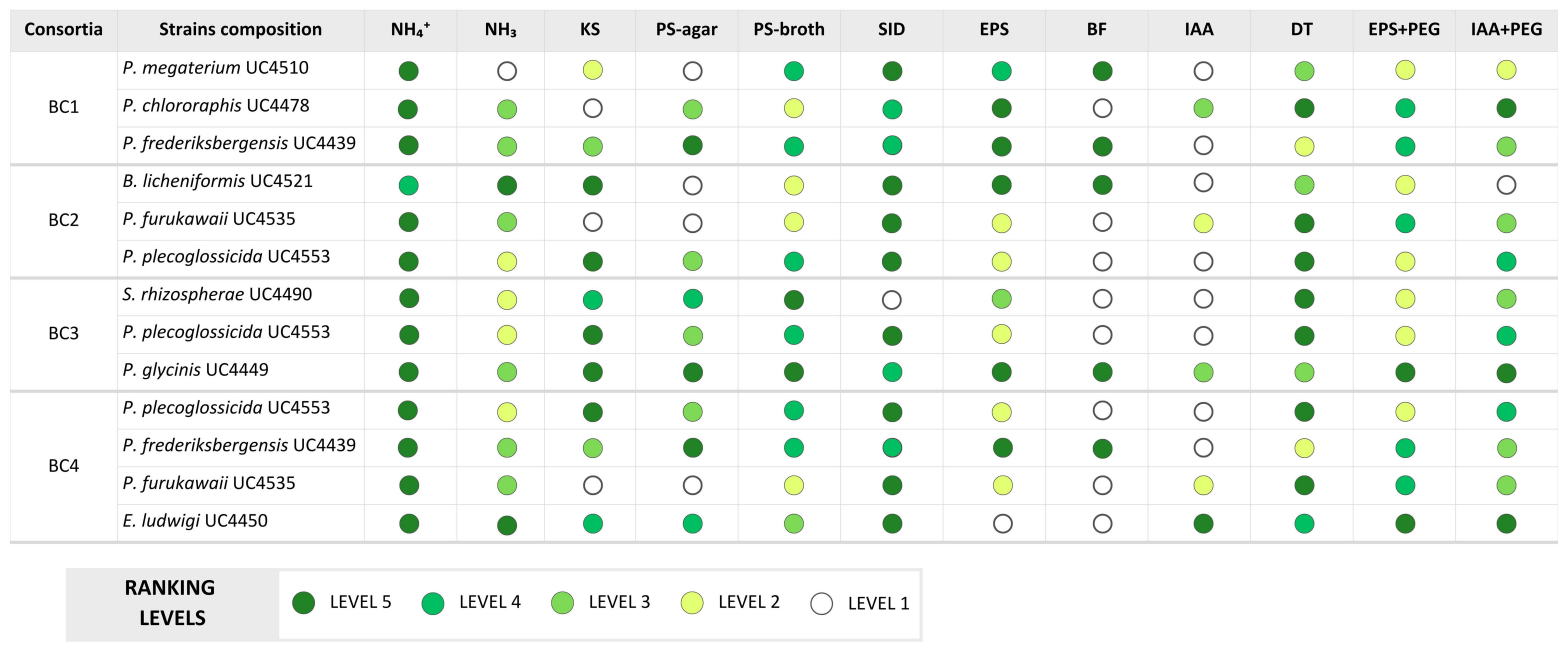
Composition of the four bacterial consortia (BC1-4) used in the pot experiment and associated PGPTs. Trait abbreviations: NH_4_^+^, NH_3_, Ammonium/Ammonia production; KS, potassium solubilization; PS-agar, phosphorous solubilization in agar; PS-broth, phosphorous solubilization in broth; SID, siderophore production; EPS, exopolysaccharides production; EPS+PEG, exopolysaccharides production in 18% of PEG; BF, Biofilm formation; IAA, Indole-3-acetic acid production; IAA+PEG, Indole-3-acetic acid production in 18% of PEG; DT, drought tolerance; assessed by OD_600_ values ranging from 0.50 to 1.00 in TSB medium supplemented with 24% PEG 6000 (-1.67 MPa).

Compatibility tests revealed antagonism between *B. licheniformis* UC4521 and four strains: *E. ludwigii* UC4450, *P. chlororaphis* UC4478, *S. rhizosphaerae* UC4490, and *P. frederiksbergensis* UC4439. Additionally, *P. megaterium* UC4510 exhibited limited growth with *P. furukawaii* UC4535, *P. plecoglossicida* UC4553 and *P. glycini*s UC4449. To avoid antagonistic interactions and limited growth, these strains were distributed across separate consortia. The remaining strains were considered mutually compatible and were grouped based on complementary PGPT profiles and potential synergistic interactions. To maximize functional diversity, strains were combined in each consortium to ensure a broad coverage of PGPTs, so that the resulting formulations could collectively express a wide range of beneficial activities. The final consortia compositions and the PGPT traits covered by each of the four BC are illustrated in **Figure 1**, where traits efficiently expressed (Level 5) by each strain are highlighted in dark green. The figure illustrates how the selected strains were strategically grouped to achieve functional complementarity across key PGPT categories.

Overall, the nine strains were selected from the highest-ranked isolates based on their quantitative PGPT profiles, excluding taxa with known pathogenic potential. These strains were then assembled into rational and manageable consortia for an initial pot-screening experiment, based on complementarity of PGPT expression, strain compatibility, and potential synergistic interactions associated with their shared origin. This strategy ensured that the most promising and biosafe candidates were evaluated while maintaining experimental feasibility in the in-planta trial.

### 3.3 Genome-based functional prediction of plant growth-promoting traits

Short-read WGS using the Illumina NovaSeq technology enabled the generation of high-quality genome assemblies for all nine selected PGPR strains. Despite the use of short reads only, all genomes assemblies obtained with Unicycler displayed satisfactory quality metrics (data not shown), supporting downstream comparative and functional genomic analyses.

To explore the genomic basis of PGPTs, the predicted protein sequences of each genome were screened using the PGPT-Pred tool available in PLaBAse (**Supplementary Table S3**). The list of PGPT-associated genes is presented in **Supplementary Table S4**, categorized into four major functional groups: N acquisition, P solubilization, siderophore production, and phytohormone regulation.

Across the nine strains, a diverse repertoire of PGPT-related genes was identified in all four categories. Within the nitrogen acquisition category, several isolates encoded key genes involved in nitrogen metabolism. These included markers of nitrification (*e.g.*, *nitABR*1, *ntrABC*), denitrification (*nirK*, *nosZ*, *nxrAB*) and urea metabolism (*ureABCDFGJ*, *urtABCDE*). A few genes related to nitrogen fixation were also found, such as *nifM*, *nifS*, *nifU*, *fixN*, *fixO*. However, the absence of structural nitrogenase genes (*nifHDK*) suggests that none of the isolates are capable of fixing atmospheric N_2_.

Genes involved in P solubilization were widespread among the strains. Notable hits included the *pho* regulon, commonly associated with inorganic phosphate uptake and metabolism. Genes coding for phosphatases were consistently found across all strains, while phytase-related genes (*phyABCD*) were uniquely identified in *B. licheniformis* UC4521, indicating a potential advantage in mobilizing organic phosphorus for this strain. Additionally, genes involved in the mobilization of other forms of P like phosphonates were found across most of the selected strains.

In the siderophore production category, multiple isolates carried genes linked to the biosynthesis and transport of iron-chelating molecules. These included operons for pyoverdine (*pvdAEQRT*) and pyochelin (*pchB*), as well as multidrug and metal transport systems (*mdtABC*), highlighting their ability to improve iron acquisition under limiting conditions, a common feature in stressed rhizospheres.

Regarding phytohormone regulation, most strains harbored genes from the *trp* cluster, involved in auxin metabolism. However, the gene *ipdC*, which catalyzes a central step in IAA biosynthesis via the indole-3-pyruvate pathway, was exclusively found in *E. ludwigii* UC4450. Additional gene hits included *gabT*, *gabD*, *aldH*, and *sad*, which are components of the GABA shunt, a stress-associated signaling and metabolic route relevant to plant-microbe interactions. Genes from the *puu* operon (*puuBCDU*) and *spuC/patD*, involved in putrescine catabolism, were also detected, suggesting roles in polyamine regulation, a mechanism increasingly recognized for its involvement in plant stress responses. Furthermore, the detection of a P450-dependent 3Fe-4S ferredoxin in *P. megaterium* UC4510, *B. licheniformis* UC4521, and *P. furukawaii* UC4535, suggests a potential role of these isolates in modulation of gibberellin like compounds via oxidative transformations.

Overall, although gene presence does not necessarily imply expression, some detected PGPT-related genes were consistent with the *in vitro* phenotypes, reinforcing the selection of these strains as robust, functionally promising members of the consortia later tested in pots.

### 3.4 Biosafety assessment through virulence factor and AMR gene screening

To assess the biosafety of the nine selected strains, genomes were screened against AMR and VF databases using ABRicate with the ResFinder, CARD, and VFDB repositories. The complete list of VF and AMR gene hits detected in each strain is reported in **Supplementary Table S5**. Whole-genome VF screening using the VFDB database revealed the presence of various genes associated with motility (*fli*, *flg*), secretion systems (*vipA/B*, *hcp1*, *clpV1*), and biofilm formation (*alg*, *pil*, *waa*) across several isolates. Notably, multiple pseudomonads, particularly *P. chlororaphis* UC4478 and *P. furukawaii* UC4535, harbored a well-represented Type VI Secretion System (T6SS) gene cluster, including *vipA/B*, *hcp1*, and *clpV1*. Additionally, genes involved in pyoverdine biosynthesis (*pvdS*, *pvdH*, *pvdA*) and alginate production (*algU*, *algI*, *algD*) were identified in several isolates, supporting their ability of abiotic stress adaptation and nutrient acquisition. The only isolate carrying genes potentially related to opportunistic human pathogenicity was *E. ludwigii* UC4450, which contained *ompA*, *csgG*, and *entB*. These genes are associated with outer-membrane integrity, fimbrial adhesion, and siderophore-mediated iron uptake in some clinical *Enterobacteriaceae*.

In parallel, the screening of AMR determinants revealed a limited set of hits across most genomes, primarily associated with intrinsic or low-risk resistance mechanisms. *Pseudomonas* isolates, including *P. chlororaphis* UC4478 and *P. furukawaii* UC4535, carried genes belonging to the *mexAB-oprM* and *mexCD-oprJ* multidrug efflux systems (e.g., *mexB*, *mexD*, *mexF*, *oprJ*, *opmH*, *cpxR*), commonly associated with basal resistance and solvent tolerance in environmental pseudomonads. These genes are ubiquitous in soil- and rhizosphere-associated *Pseudomonas* species and are not considered clinically relevant in the absence of mobile genetic elements or co-localized β-lactamases, which were not detected. Conversely, *Enterobacter ludwigii* UC4450 carried a broader AMR gene repertoire, including *bla*ACT-12 (class C β-lactamase) with full coverage and high identity (100 %, 98.8 %), consistent with AmpC-type resistance typically reported in the *E. cloacae* complex. Additional resistance determinants included *FosA2* (fosfomycin resistance), the efflux pump genes *oqxA/B*, *acrAB*, and *mdtC*, and global regulators (*marA*, *ramA*, *cpxA*, *CRP*), which together could contribute to reduced susceptibility to multiple antibiotic classes in clinical contexts. The genomic localization and mobility of AMR genes should be verified in future work using long-read sequencing approaches to ensure full biosafety compliance prior to potential large-scale applications.

Overall, the genomic screening revealed mainly intrinsic and low-risk AMR and VF profiles. Although *E. ludwigii* UC4450 displayed a broader set of AMR and VF genes, these features remain acceptable within the context of a controlled experimental screening and do not preclude its use for preliminary in-planta evaluation.

### 3.5 In planta evaluation of physiological performances and biomass quantification

**Figure 2:**
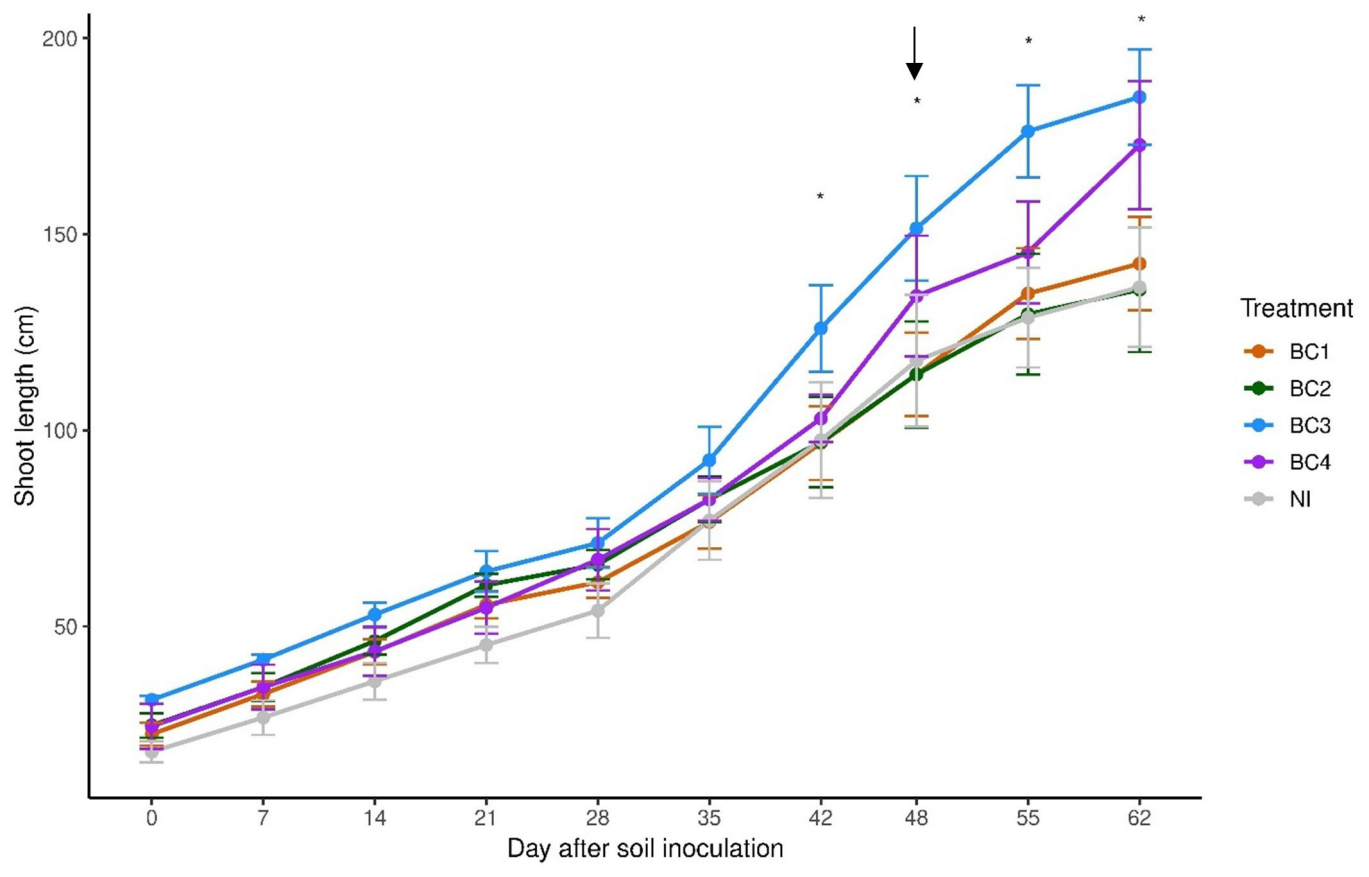
Mean total shoot length (sum of two shoots) of one-year-old *Vitis vinifera* L. across different consortia treatments, measured over a 62-day period following the second inoculation. Black arrow indicates the start of dry-down water stress. Error bars represent the standard error of the mean. *indicate significant differences between treatments according to Fisher’s LSD *post hoc* test following a significant ANOVA (p< 0.05).

**Figure 3:**
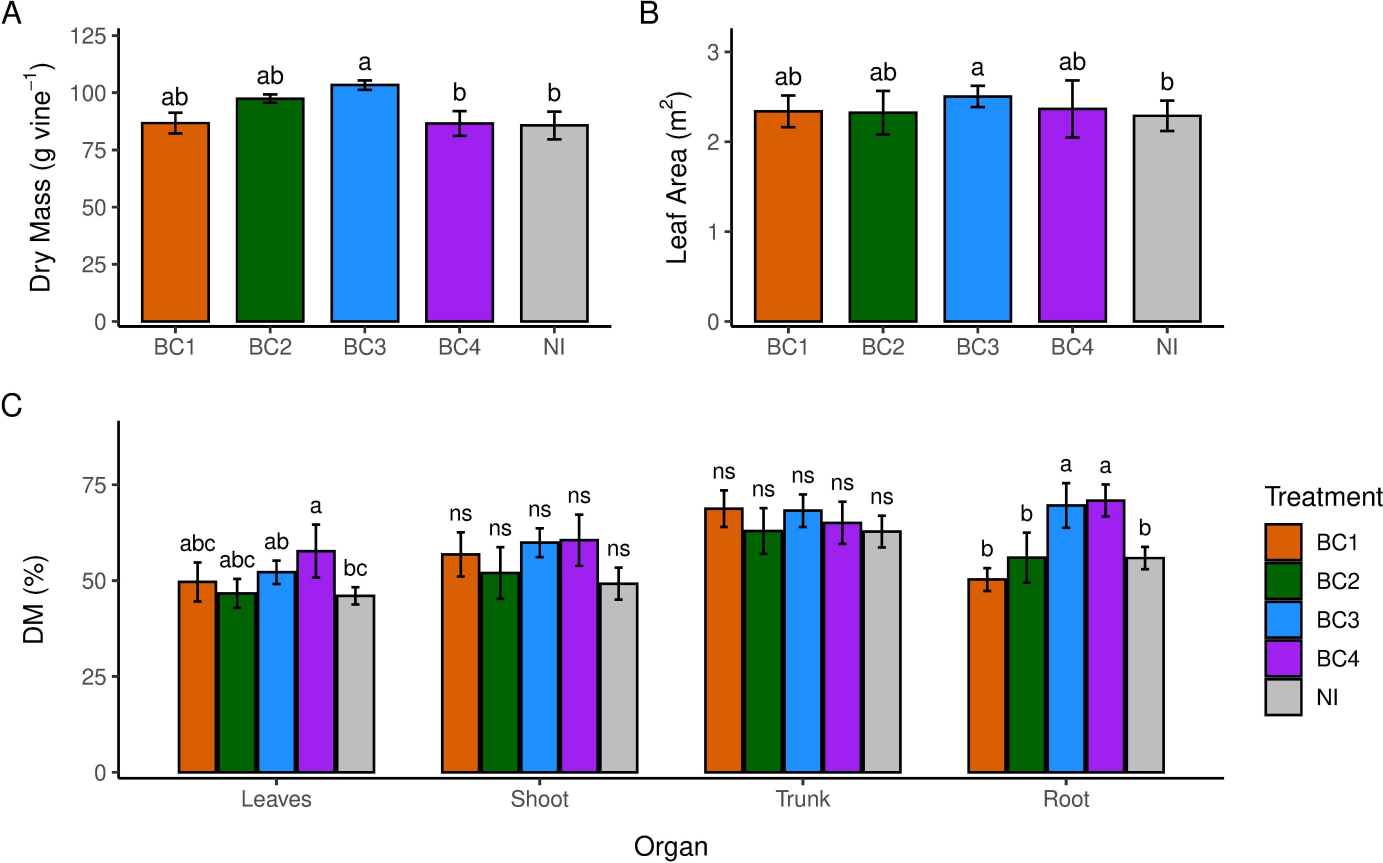
Total Dry Mass **(A)**, Leaf Area **(B)** and percentage of Dry Matter (DM) **(C)** allocated to grapevines organs at the end of the experiment across consortia treatments (BC1, BC2, BC3, BC4) and non-inoculated control (NI). Bars indicate standard error bars of the mean. Within each compartment, different letters indicate significant differences at p< 0.05 according to Fisher’s LSD test following a significant ANOVA; ns, not significant.

No significant differences in vegetative growth, expressed as the sum of the two shoots per vine, were observed among treatments during the first 35 days following the second inoculation. However, differences emerged starting from day 42 (**Figure 2**). BC1 and BC2 did not enhance grapevine vegetative growth compared to the non-inoculated control (NI). In contrast, consortia BC3 and BC4 significantly improved growth. Specifically, BC3 exhibited earlier and higher shoot growth during the initial phase; however, growth rate slew after day 55. Conversely, BC4 showed similar growth to BC1, BC2, and NI until 55 days after soil inoculation, after which it exhibited a markedly higher shoot elongation than all other treatments. In treatments BC1, BC2, BC3, and NI, shoot growth reached a plateau during the drought period (days 55-62). No physiological differences were observed under full irrigation or at 60% of ET (**Supplementary Table S6**). On the 2nd day of completed suspended irrigation (**Table 3**, June 29^th^), no difference was found among treatments in terms of leaf photosynthetic rates. However, BC3 showed a reduced stomatal conductance compared to other treatments (approximately half), resulting in higher instantaneous water use efficiency. On the 5th day with no irrigation (**Table 3**, July 2^nd^), a marked decline in photosynthetic rates was observed in all treatments, with values dropping below 2 μmol CO_2_ m^-^² s^-^¹. Only BC4 vines maintained a higher photosynthetic rate (4.33 μmol m^-^² s^-^¹ vs an average of 1.12 μmol m^-^² s^-^¹ of the other pooled treatments) along with higher stomatal conductance (0.06 mol m^-^² s^-^¹), while all the other treatments exhibited almost complete stomatal closure. Despite sustained photosynthetic activity, Ψ_MD_ in BC4 did not significantly differ from the other treatments. Conversely, BC3 displayed earlier stomatal closure (**Table 3**, June 29^th^) and reached a pre-dawn leaf water potential of -1.2 MPa, while the other treatments maintained values above -1.0 MPa (**Table 3**, July 2^nd^). At the end of the trial, BC3 vines exhibited a higher total dry mass compared to the other treatments and a significantly larger leaf area than NI (**Figure 3**). Both BC3 and BC4 showed greater shoot length and significantly higher root biomass than BC1, BC2, and NI.

**Table 3:** Physiological parameters recorded during the drought period across consortia treatments (BC1-4) and non-inoculated control (NI).

Treatment	Leaf E^a^	Leaf gs^a^	Leaf A^a^	Leaf Ψ_MD_^a^	Leaf Ψ_PD_^a^
June 29^th b^					
BC1	2.67	0.136b	9.01	-0.94	-0.79
BC2	2.53	0.113b	6.93	-1.01	-0.74
BC3	1.44	0.050a	6.88	-0.90	-0.70
BC4	2.78	0.136b	9.44	-0.82	-0.92
NI	2.93	0.130b	7.44	-0.76	-0.84
Sign.	ns	*	ns	ns	ns
July 2^nd b^					
BC1	0.13b	0.000b	1.10b	-1.06b	-0.78b
BC2	0.12b	0.007b	0.66b	-1.08ab	-0.77b
BC3	0.00b	0.000b	1.78b	-1.35a	-1.16a
BC4	2.00a	0.060a	4.33a	-1.17ab	-0.78b
NI	0.22b	0.013b	0.97b	-1.10ab	-1.00ab
Sign.	*	***	*	*	*

Mean separation was performed using Fisher’s LSD test when the ANOVA F test was significant; different letters indicate significant differences (*p<0.05, ***p<0.001, ns, not significant. ^a^Leaf E =Leaf transpiration rates (mmol m^-2^ s^-1^), Leaf gs =Leaf stomatal conductance (mol m^-2^ s^-1^), Leaf A =Leaf photosynthetic rates (μmol m^-2^ s^-1^), Leaf Ψ_MD_ =midday water potential (MPa); Leaf Ψ_PD_ =pre-dawn water potential (MPa). ^b^June 29^th^ = second day of suspended irrigation and July 2^nd^ = fifth day of suspended irrigation.

## 4 Discussion

In this study, we explored the potential of drought-tolerant PGPRs isolated from the rhizosphere of grapevines previously exposed to drought stress, under the hypothesis that plants may actively modulate their microbiome to recruit beneficial microorganisms in response to adverse conditions (Cesco et al., 2021; Rolfe et al., 2019). We adopted a comprehensive experimental pipeline involving the isolation, phenotypic and genotypic characterization of drought-adapted strains, the formulation of consortia with complementary PGPTs, and their *in vivo* evaluation through a pot trial on one-year-old grapevines subjected to progressive drought stress, where vegetative and physiological parameters were monitored to infer their growth-promoting capacity and potential physiological response under water deficit. This approach revealed two BC with different temporal effects: one stimulated early growth under non-limiting conditions, while the other showed stronger effects during water deficit, suggesting distinct application potentials.

In our study, most isolates belonged to well-known PGPR such as *Bacillus* and *Pseudomonas*, which are recognized for their ability to alleviate abiotic stress by modulating phytohormone levels and enhancing antioxidant defense systems (Dagher et al., 2025). However, since the beneficial and safe behavior of a microorganism cannot be assumed based solely on its genus or species, accurate taxonomic identification must be complemented with strain-level functional characterization and genome-based biosafety screening before considering field application.

Consistent with our findings, other studies have shown that PGPR applications can enhance plant drought tolerance by modulating stress-related gene expression and improving physiological responses (Belimov et al., 2009; Cho et al., 2013; Kulkova et al., 2024; Lim and Kim, 2013; Rashid et al., 2022; Yasmin et al., 2022).

Microorganisms naturally develop surface-attached biofilms (Flemming and Wingender, 2010). In agriculture, biofilm-forming PGPR improve drought tolerance by enhancing water retention, root colonization, and rhizosphere overall moisture (Bhattacharyya et al., 2021; Li et al., 2024). In our study, *E. ludwigii* UC4450, *P. chlororaphis* UC4478, and *S. rhizosphaerae* UC4490 showed biofilm-forming ability. As in most studies, this was assessed at the strain level; however, multispecies interactions can markedly alter biofilm structure and function, an aspect worth exploring further.

The selected strains showed different expression of stress-related PGPTs, such as EPS and IAA, under simulated drought conditions *in vitro*. This indicates that the strains can adapt to water-limited environments, although their responses vary depending on the specific trait. All isolates produced higher levels of EPS when exposed to reduced water potential, suggesting that EPS synthesis is part of their adaptive strategy and may enhance bacterial survival and root colonization (Ali et al., 2014). In contrast, IAA production, a hormone regulating root development and stress tolerance, showed a more variable pattern. Most strains maintained or slightly increased IAA levels under osmotic stress whereas others exhibited a marked decrease, confirming that phytohormone synthesis is strain-dependent and sensitive to environmental conditions. For instance, *E. ludwigii* UC4450 produced high amounts of IAA under optimal conditions but showed a 48% decrease under osmotic stress, a trend also reported for *Pseudomonas putida* under reduced osmotic potential (Sandhya et al., 2009), suggesting that IAA synthesis is commonly downregulated by osmotic stress. These findings highlight the functional diversity among PGPR and emphasize the importance of evaluating their performance under both optimal and stress conditions. Overall, these results highlight the functional diversity among PGPR and suggest that adaptive traits may contribute to their persistence and to plant resilience under drought (Glick, 2012).

Since no fertilizer was applied during the experiment, nutrient-mobilizing traits previously identified in these strains, such as phosphorus and potassium solubilization or siderophore production, may have indirectly supported plant growth. BC3 promoted early shoot growth under well-watered conditions, suggesting that this consortium is effective in stimulating vegetative development independently of stress. In contrast, the beneficial effects of BC4 became progressively evident as drought stress intensified, indicating that its constituent strains may be specifically activated by water deficit and express their growth-promoting functions more effectively under limiting conditions. This contrast highlights distinct modes of action among the consortia, with BC3 acting as a general growth enhancer and BC4 exhibiting traits more closely associated with drought stress mitigation. The latter observation aligns with previous findings by (Rolli et al., 2015), who demonstrated that many PGPR traits are conditionally expressed and become more evident under stress rather than being constitutively active.

Although individual strains were not tested in this study, the differential performance of microbial consortia suggests that not all combinations are equally effective. No significant differences observed in BC1 and BC2, both containing *Bacillus* and *Pseudomonas* strains, may reflect limited functional synergy among the inoculated members or possible antagonistic interactions with native soil microorganisms under natural environmental conditions (Trabelsi and Mhamdi, 2013). This is consistent with previous findings showing that microbial mixtures can underperform due to competition or incompatibility among constituent strains (Finkel et al., 2017; Rolli et al., 2015). For instance, since consortium BC2 included *P. plecoglossicida* UC4553 and *P. furukawaii* UC4535, both also present in the more effective BC4, together with *Bacillus licheniformis* UC4521, it is possible that the latter exerted a negative influence on the co-inoculated strains and the native soil microbiota, potentially limiting microbial establishment and the expression of beneficial traits (Trabelsi and Mhamdi, 2013).

Although *ompA*, *csgG*, and *entB* are linked to virulence, and *bla*ACT-12 and *Fos*A2 to antimicrobial resistance in some *Enterobacteriaceae*, the presence of these genes in our environmental strains *E*. *ludwigii* UC4450 likely reflects ecological adaptation. Such strains are typically less concerning than clinical isolates (Mishra et al., 2020), although thorough biosafety assessment remains essential before considering their commercial use. The contribution of *Enterobacter* is particularly relevant, as its role in enhancing grapevine drought resilience has been previously documented (Rolli et al., 2015) and may help explain the increased photosynthetic activity observed in BC4. This interpretation is supported by reports showing that *Enterobacter* sp. can increase stomatal conductance by 44% in drought-stressed maize (Naveed et al., 2014). The superior performance of BC4 may thus result from synergistic effects among the three *Pseudomonas* strains (*P. plecoglossicida* UC4553, *P. frederiksbergensis* UC4439, and *P. furukawaii* UC4535) and *E. ludwigii* UC4450, all of which, under osmotic stress, produced high levels of EPS and were among the highest siderophore producers, traits that enhance iron acquisition and contribute to drought resilience (Arzanesh et al., 2011; Rajkumar et al., 2017). Such co-production of siderophores and IAA may have further supported plant growth, helping plants remain metabolically active during water deficit, while the absence of this trait in some strains, like *S. rhizosphaerae* UC4490 in BC3, could partially limit their functional potential under water-limited conditions. However, as mentioned above, BC3 performed better under well-watered conditions, which may be related to its strain composition. In particular, the consortium included *P. glycinis* UC4449, one of the isolates showing the highest overall PGPT potential, including strong *in vitro* IAA production. This strain was combined with *S. rhizosphaerae* UC4490 and *P. plecoglossicida* UC4553, which also exhibited IAA production together with other PGPTs, such as NH_3_ production, phosphate and potassium solubilization. Under a non-fertilized substrate, the combined expression of these traits may have contributed to enhanced vegetative growth.

With the application of BC3, early shoot growth resulted in a larger leaf area and likely higher water consumption, reducing the amount of water available during irrigation withholding and leading to lower Ψ_PD_ values at the end of the trial. The rapid decline in gs to nearly zero after two days of water suspension suggests a fast defensive regulatory response, likely triggered by reduced soil water availability compared with the other treatments. This response is consistent with a growth-oriented strategy promoted by BC3 under non-limiting water conditions, which may predispose plants to earlier stomatal closure once water becomes limiting. Under optimal conditions, such as in irrigated vineyards or regions with a stable water supply, the early vigor observed in BC3 could still represent a growth advantage. In contrast, BC4 maintained active gas exchange and photosynthetic activity during water deficit, resulting in greater final plant growth.

Finally, the greater shoot length and root biomass observed in BC3 and BC4 may contribute to more uniform and vigorous development in the following season. This is particularly relevant during the early stages of vineyard establishment, as faster root and canopy development reflects better adaptation to the surrounding environment and may shorten the unproductive phase, thus accelerating the onset of productivity (Reynolds and Heuvel, 2009). At this stage, young vines also offer a favorable window for microbial colonization and benefit expression, whereas in adult vines, the presence of a well-established microbiome and a more complex root system may limit the effectiveness of inoculation, requiring alternative strategies in terms of application timing, formulation, or delivery method.

## 5 Conclusions

Climate change and increasingly unpredictable weather events are challenging viticultural production, particularly in semi-arid regions where drought critically limits vine growth and productivity. Developing sustainable and biologically based strategies to mitigate these effects is therefore a key priority for viticulture. Our results demonstrate that an integrated pipeline for isolating, screening, characterizing, and rational assembly of drought-tolerant PGPR strains was effective in developing two potential functional microbial consortia that enhanced the growth of young grapevines during early developmental stages. Among the tested consortia, BC3 and BC4, significantly promoted shoot and root development in young grapevines, albeit with distinct response patterns. The observed effects are likely attributable to synergistic interactions among compatible strains or the dominance of specific PGPRs expressing key functional traits. BC3 stimulated early shoot growth under well-watered conditions, whereas BC4 led to a marked increase in shoot elongation and better maintenance of physiological performance under water-limited conditions. These results highlight the potential of PGPR-based consortia as promising tools to improve young grapevine growth under both optimal and drought conditions.

Future research should focus on validating these effects at the field scale and over longer time frames, including subsequent productive seasons. In parallel, further work is required to improve formulation stability, assess the persistence and colonization dynamics of the inoculated strains, and refine strain-function relationships through targeted functional analyses. From an applied perspective, such efforts are essential to translate PGPR-based strategies into reliable and scalable tools for vineyard management under increasing water scarcity.

## Data Availability

The genomic data generated in this study have been deposited in the National Center for Biotechnology Information (NCBI) repository under accession number PRJNA1404239.
